# Anti-neuroinflammatory effects of cudraflavanone A isolated from the chloroform fraction of *Cudrania tricuspidata* root bark

**DOI:** 10.1080/13880209.2018.1447972

**Published:** 2018-03-09

**Authors:** Kwan-Woo Kim, Tran Hong Quang, Wonmin Ko, Dong-Cheol Kim, Chi-Su Yoon, Hyuncheol Oh, Youn-Chul Kim

**Affiliations:** a College of Pharmacy, Wonkwang University, Iksan, Republic of Korea;; b Institute of Marine Biochemistry, Vietnam Academy of Science and Technology (VAST), Hanoi, Vietnam;; c Hanbang Cardio-Renal Syndrome Research Center, Wonkwang University, Iksan, Republic of Korea

**Keywords:** BV2 microglial cell, neuroinflammation, nuclear factor kappa B, mitogen-activated protein kinase, heme oxygenase-1, nuclear transcription factor erythroid-2 related factor 2

## Abstract

**Context:***Cudrania tricuspidata* Bureau (Moraceae) is an important source of traditional Korean and Chinese medicines used to treat neuritis and inflammation.

**Objective:** The anti-neuroinflammatory effects of cudraflavanone A isolated from a chloroform fraction of *C. tricuspidata* were investigated in LPS-induced BV2 cells.

**Materials and methods:** Cudraflavanone A was isolated from the root of *C. tricuspidata*, and its structure was determined by MS and NMR data. Cytotoxicity of the compound was examined by MTT assay, indicating no cytotoxicity at 5–40 μM of cudraflavanone A. NO concentration was measured by the Griess reaction, and the levels of PGE_2_, cytokines and COX-2 enzyme activity were measured by each ELISA kit. The mRNA levels of cytokines were analysed by quantitative-PCR. The expression of iNOS, COX-2, HO-1, NF-κB, MAPKs and Nrf2 was detected by Western blot.

**Results:** Cudraflavanone A had no major effect on cell viability at 40 μM indicating 91.5% viability. It reduced the production of NO (IC_50_ = 22.2 μM), PGE_2_ (IC_50_ = 20.6 μM), IL-1β (IC_50_ = 24.7 μM) and TNF-α (IC_50_ = 33.0 μM) in LPS-stimulated BV2 cells. It also suppressed iNOS protein, IL-1β and TNF-α mRNA expression. These effects were associated with the inactivation of NF-κB, JNK and p38 MAPK pathways. This compound mediated its anti-neuroinflammatory effects by inducing HO-1 protein expression via increased nuclear translocation of Nrf2.

**Discussion and conclusions:** The present study suggests a potent effect of cudraflavanone A to prevent neuroinflammatory diseases. Further investigation is necessary to elucidate specific molecular mechanism of cudraflavanone A.

## Introduction

Neuroinflammation is generally associated with neurodegenerative disorders of the central nervous system (CNS) (Groh and Martini 2017). Microglia are the resident macrophages of the CNS, and play significant roles in homeostasis and neuroinflammatory pathologies in the brain (Cianciulli et al. 2016). The activation of microglial cells is known to play a significant role in neuroinflammation through the excessive production of pro-inflammatory mediators (Okorji et al. 2016). Under inflammatory conditions, microglial cells may get activated and cause abnormal regulation of pro-inflammatory mediators, such as nitric oxide (NO), prostaglandin E2 (PGE_2_), inducible nitric oxide synthase (iNOS), cyclooxygenase (COX)-2 and pro-inflammatory cytokines as like interleukin (IL)-1β, IL-6 and tumour necrosis factor (TNF)-α (Streit et al. 2004; More et al. 2013). This series of inflammatory reactions is related to the activation of nuclear factor κ-light-chain-enhancer of activated B cells (NF-κB) and mitogen-activated protein kinase (MAPK) pathways (Baliga et al. 2012). These pathways are activated by various stimuli, and result in the up-regulation of NF-κB and MAPK target genes, as observed with pro-inflammatory mediators (Lawrence 2009; Velagapudi et al. 2014). Therefore, the regulation of inflammation in the nervous system mediated by microglial cell is related to the various pathogenesis of neurodegenerative diseases, including Alzheimer’s disease (AD), Parkinson’s disease (PD), frontotemporal dementia and amyotrophic lateral sclerosis (Catorce and Gevorkian 2016).

Heme oxygenase (HO)-1 could be induced in response to immunological activities as like inflammation or oxidative stress. HO-1 is regulated by the nuclear transcription factor erythroid-2 related factor 2 (Nrf2) pathway. Several reports have demonstrated that the activation of HO-1 signalling results in the inhibition of inflammatory responses (Matz et al. 1996; Lin et al. 2014). Therefore, HO-1 is one of the potential targets for the treatment of various inflammatory diseases.

*Cudrania tricuspidata* Bureau (Moraceae) is a deciduous plant used as a traditional medicinal herb for the treatment of various disorders (Zhang 1985; Xin et al. 2017), as this plant is a rich source of bioactive compounds with antioxidant, neuroprotective and anti-inflammatory effects (Lee et al. 2005; Park et al. 2006; Kwon et al. 2014). Recent studies have highlighted the various components isolated from *C. tricuspidata* with anti-inflammatory effects (Jo et al. 2017; Tuan Anh et al. 2017). Our previous report showed that the compounds isolated from this plant display anti-neuroinflammatory effects (Yoon et al. 2016). Cudraflavanone A is one of the components contained in *C. tricuspidata*, and it has been reported that cudraflavanone A inhibits topoisomerase I and protein kinase C (PKC) activity leading to inducing the apoptotic cell death of human cancer cells (Rho et al. 2007), and vascular smooth muscle cells growth via an Akt-dependent pathway (Han et al. 2007). However, the anti-neuroinflammatory effects of cudraflavanone A on microglial cells have not been investigated yet. Therefore, as a part of our ongoing searching for novel compounds from natural products to treat, the present study describes the isolation of cudraflavanone A from *C. tricuspidata* and its potent effect as an anti-neuroinflammatory agent in BV2 cells.

## Materials and methods

### Plant materials

The root barks of *C. tricuspidata* were purchased in May 2014 from Daerim Korean crude drug store, Kumsan, Chungnam Province, Korea, and identified by Dr. Kyu-Kwan Jang, Botanical Garden, Wonkwang University. A voucher specimen (WP-2014-12) was deposited at the Herbarium of the College of Pharmacy, Wonkwang University (Iksan, Korea). Cudraflavanone A was isolated from the chloroform fraction of the methanol extract of *C. tricuspidata* by various chromatographic methods. The structure of the compound was determined by mass spectrometry (MS) and nuclear magnetic resonance (NMR) analysis (Quang et al. 2015).

### Chemicals and reagents

Dulbecco’s modified Eagle’s medium (DMEM), foetal bovine serum (FBS), and other tissue culture reagents were purchased from Gibco BRL Co. (Grand Island, NY). All other chemicals, including lipopolysaccharide (LPS), cobalt protoporphyrin IX chloride (CoPP) and 3-(4,5-dimethylthiazol-2-yl)-2,5-diphenyltetrazolium bromide (MTT), were obtained from Sigma-Aldrich (St. Louis, MO). Primary antibodies such as anti-iNOS, anti-COX-2, anti-inhibitor kappa B (IкB)-α, anti-p-IкB-α, anti-p50, anti-p65, anti-actin and anti-proliferating cell nuclear antigen (PCNA) antibodies were purchased from Santa Cruz Biotechnology (Dallas, TX). Anti-p-extracellular signal-regulated kinase (ERK), anti-ERK, anti-p-c-Jun N-terminal kinase (JNK), anti-JNK, anti-p-p38, anti-p38, anti-p-protein kinase B (Akt) and anti-Akt were obtained from Cell Signaling Technology (Danvers, MA). Anti-HO-1 antibody was gained from Merck Millipore (Darmstadt, Germany), and anti-Nrf2 antibody was purchased from Abcam (Cambridge, MA). Anti-mouse, anti-goat and anti-rabbit secondary antibodies were supplied by Merck Millipore (Darmstadt, Germany). Tin protoporphyrin IX (SnPP IX), an inhibitor of HO activity, was obtained from Porphyrin Products (Logan, UT). The enzyme-linked immunosorbent assay (ELISA) kit for PGE_2_ was purchased from R&D Systems, Inc. (Minneapolis, MN).

### Cell culture

BV2 cells were maintained at 5 × 10^6^ cells/dish and 5 × 10^5^ cells/mL in a 100 mm dish in diameter in DMEM supplemented with 10% (v/v) heat-inactivated FBS, penicillin G (100 units/mL), streptomycin (100 μg/mL) and l-glutamine (2 mM), and incubated at 37 °C in a humidified atmosphere containing 5% CO_2_.

### Cell viability analysis with MTT assay

Cell viability was determined by using the MTT assay as previously described (Ngan et al. 2017). The assay was conducted three times, independently.

### Western blot analysis

The details of Western blot analysis are described in the previous report (Ngan et al. 2017). Briefly, BV2 cells were harvested and pelleted by centrifugation at 16,000×*g* for 15 min. The cells were washed with phosphate-buffered saline (PBS) and lysed with 20 mM Tris–HCl buffer (pH 7.4) containing a protease inhibitor mixture (0.1 mM phenylmethylsulphonylfluoride (PMSF), 5 mg/mL aprotinin, 5 mg/mL pepstatin A and 1 mg/mL chymostatin). An equal amount of protein from each sample was resolved using 7.5% and 12% sodium dodecyl sulphate-polyacrylamide gel electrophoresis (SDS-PAGE).

### Determination of nitrite (NO production)

The nitrite concentration in the medium, an indicator of NO production, was measured by the Griess reaction, as previously described (Ngan et al. 2017).

### PGE_2_ assay

The level of PGE_2_ present in each sample was determined using a commercially available kit from R&D Systems (Minneapolis, MN). Three independent assays were performed according to the manufacturer's instructions.

### Assays for IL-1β, IL-6 and TNF-α

The culture media were collected to determine the levels of IL-1β, IL-6 and TNF-α present in each sample using appropriate ELISA kits (R&D Systems, Inc., Minneapolis, MN), as per the manufacturer’s instructions.

### Quantitative real-time reverse transcription polymerase chain reaction (qRT-PCR)

Total RNA was isolated from the cells using Trizol (Invitrogen, Carlsbad, CA), according to the manufacturer’s recommendations, and spectrophotometrically quantified (at 260 nm wavelength). Total RNA (1 mg) was reverse transcribed using the High Capacity RNA-to-cDNA kit (Applied Biosystems, Carlsbad, CA). The cDNA was amplified with SYBR Premix Ex Taq kit (TaKaRa Bio, Shiga, Japan) using a StepOnePlus Real-Time PCR system (Applied Biosystems, Foster City, CA). Briefly, each 20 mL of the reaction mixture contained 10 mL of SYBR Green PCR Master Mix, 0.8 mM of each primer, and diethyl pyrocarbonate (DEPC) treated water. The primer sequences were designed using PrimerQuest (Integrated DNA Technologies, Cambridge, MA). The sequences of primers used in this experiment are shown in Table 1. The optimum conditions for PCR amplification of cDNA were established by following the manufacturer’s instructions. The data were analysed using StepOne software (Applied Biosystems, Foster City, CA) and the cycle number at the linear amplification threshold (Ct) values for the endogenous control gene (*GAPDH*) and the target gene was recorded. Relative gene expression (target gene expression normalized to the expression of the endogenous control gene) was calculated by using the comparative Ct method (2^–ΔΔCt^). The analysis was conducted three times, independently.

**Table 1.** Primers used for qPCR.

Gene	Forward primer (5′ → 3′)	Reverse primer (3′ → 5′)
IL-1β	AATTGGTCATAGCCCGCACT	AAGCAATGTGCTGGTGCTTC
IL-6	ACTTCACAAGTCGGAGGCTT	TGCAAGTGCATCATCGTTGT
TNF-α	CCAGACCCTCACACTCACAA	ACAAGGTACAACCCATCGGC
GAPDH	TTCACCACCATGGAGAAGGC	GGCATGGACTGTGGTCATGA

### Assay for COX colorimetric inhibitor screening

The COX inhibition assay was performed with colorimetric COX (Ovine) inhibitor screening assay kit (Cayman Chemical, Ann Arbor, MI), according to the manufacturer’s instructions. The assay was conducted three times, independently.

### Analysis of DNA binding activity of Nrf2

The DNA-binding activity of Nrf2 in nuclear extracts was measured using Nrf2 transcription factor (CAYMAN, Ann Arbor, MI) assay kits, according to the manufacturer’s instructions. Three independent replicates were performed.

### Statistical analysis

The data are expressed as the mean ± standard deviation (SD) of at least three independent experiments. To compare three or more groups, one-way analysis of variance (ANOVA) was used, followed by Tukey’s multiple comparison tests. The statistical analysis was performed with GraphPad Prism software, version 3.03 (GraphPad Software Inc., San Diego, CA).

## Results

### Structure and effects of cudraflavanone A on BV2 cell viability

**Figure 1.**
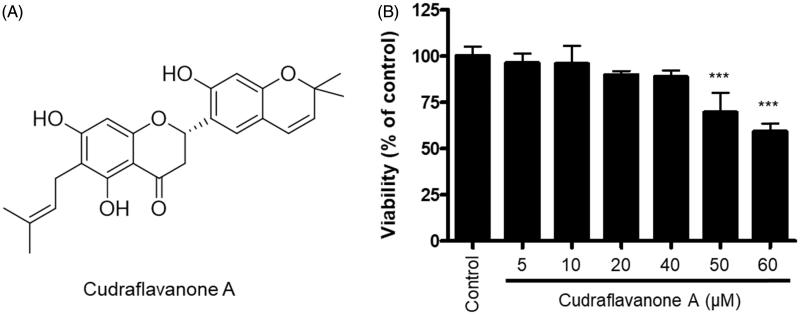
Chemical structure of cudraflavanone A (A), and effect of this compound on BV2 cell viability (B). Cells were treated with cudraflavanone A at the indicated concentrations for 24 h, and cell viability was determined by MTT assay. ***p < 0.001 in comparison with control.

The structure of cudraflavanone A was identified in the previous study (Quang et al. 2015), as shown in Figure 1(A). To evaluate the cytotoxic effects of cudraflavanone A, we determined the cell viability of BV2 cells following treatment with cudraflavanone A for 24 h at concentrations ranging from 5 to 60 μM. An MTT assay was conducted to determine optical density. Results of the MTT assay showed that cudraflavanone A at 5–40 μM concentration had no effect on BV2 cell viability (Figure 1(B)).

### Cudraflavanone A inhibited LPS-induced production of NO and PGE_2_, expression of iNOS protein, but had no effect on COX-2 in BV2 cells

**Figure 2.**
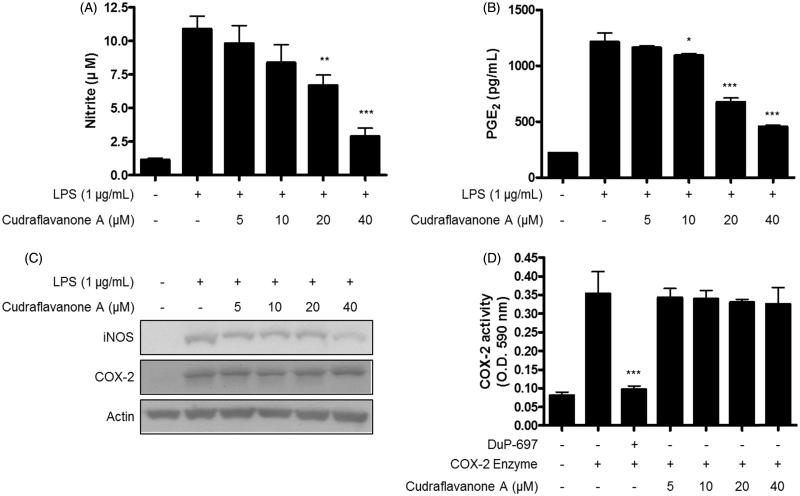
Effects of cudraflavanone A on LPS-induced NO, PGE2 production, iNOS, COX-2 protein expression and COX-2 enzyme activity in BV2 cells. (A–C) Cells were pretreated with/without the indicated concentrations of cudraflavanone A for 3 h and then stimulated with LPS (1 µg/mL) for 24 h. Nitrite levels (A) were determined using the Griess reaction and PGE2 (B) was quantified by ELISA. *p < 0.05, **p < 0.01, and ***p < 0.001 in comparison with LPS-treated group. (C) iNOS and COX-2 protein expression was determined by Western blot analysis. Representative blots from three independent experiments are shown. (D) COX-2 enzyme was treated with the indicated concentrations of cudraflavanone A for 5 min. DuP-697 was used as positive COX-2 inhibitor controls. ***p < 0.001 in comparison with COX-2 enzyme treated group. Values shown are means ± SD of three independent experiments.

The inhibitory effects of cudraflavanone A on LPS-induced production of NO and PGE_2_ were tested. BV2 cells were pretreated with or without cudraflavanone A at a nontoxic concentration range for 3 h, and stimulated by LPS (1 μg/mL) for 24 h. As a result, cudraflavanone A significantly inhibited NO and PGE_2_ production manner, with IC_50_ values 22.2 and 20.6 μM for NO and PGE_2_, respectively (Figure 2(A,B)). Continuously, the effects of cudraflavanone A on LPS-induced expression of iNOS and COX-2 protein in BV2 cells were investigated by Western blot analysis. Cudraflavanone A suppressed iNOS expression, however, it had no effect on COX-2 expression (Figure 2(C)). In addition, COX enzyme activity assay was conducted to determine the effect of cudraflavanone A on COX enzyme activity, and found that cudraflavanone A showed no effect on COX enzyme activity (Figure 2(D)).

### Cudraflavanone A inhibited the LPS-induced production of pro-inflammatory cytokines and the expression of those mRNA in BV2 cells

**Figure 3.**
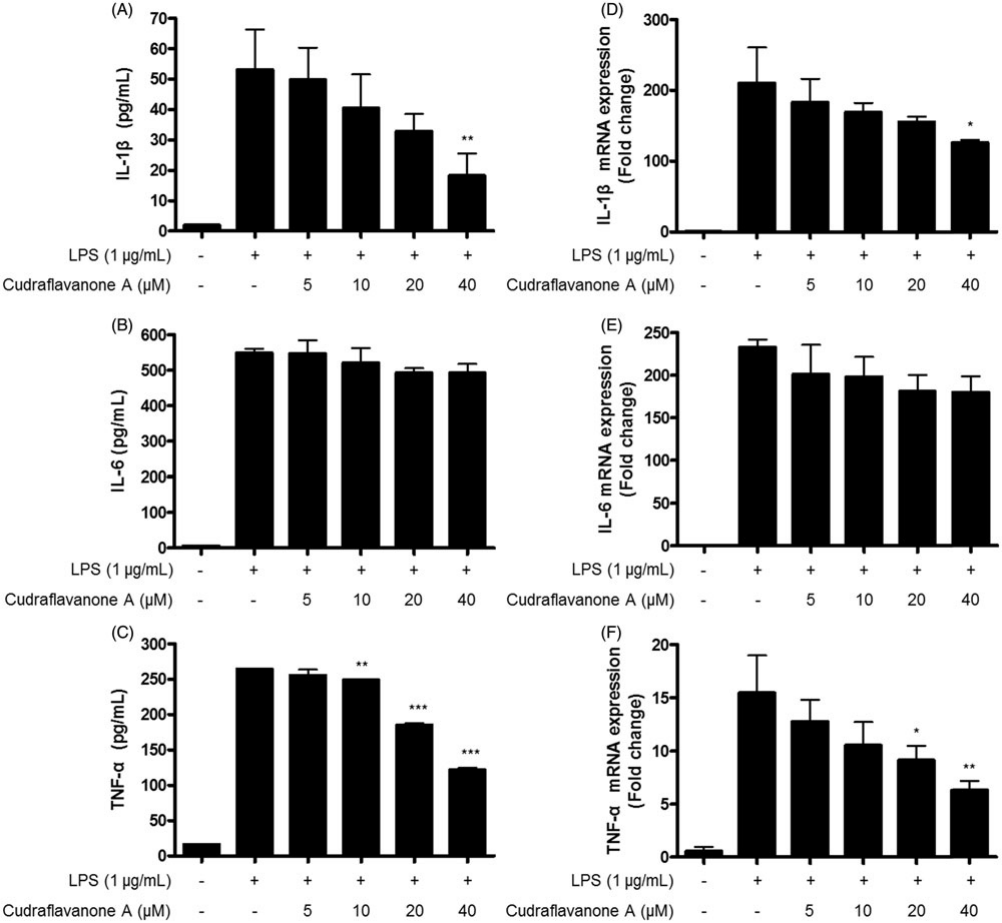
Effects of cudraflavanone A on IL-1β (A, D), IL-6 (B, E) and TNF-α (C, F) in LPS-stimulated BV2 cells. Cells were pretreated with/without the indicated concentrations of cudraflavanone A for 3 h and then stimulated with LPS (1 μg/mL) for 24 h (A–C) and 6 h (D–F), respectively. The concentration of cytokines was measured by ELISA, and mRNA expression was analysed by qPCR. *p < 0.005, **p < 0.01 and ***p < 0.001 in comparison with LPS-treated group.

Continuously, effects of cudraflavanone A on LPS-induced pro-inflammatory cytokines, as like IL-1β, IL-6 and TNF-α were examined by ELISA kits and qRT-PCR in BV2 cells. The production of IL-1β, IL-6 and TNF-α, and their corresponding mRNA expression was increased in response to LPS. However, pretreatment of cudraflavanone A decreased the production of these molecules. The reported IC_50_ values were 24.7 and 33.0 μM for IL-1β (Figure 3(A)) and TNF-α (Figure 3(C)), respectively. The mRNAs expression of IL-1β and TNF-α was also repressed by cudraflavanone A (Figure 3(D,F)). However, cudraflavanone A failed to affect the protein and mRNA levels of IL-6 (Figure 3(B,E)).

### Cudraflavanone A inhibited LPS-induced activation of the NF-κB pathway in BV2 cells

**Figure 4.**
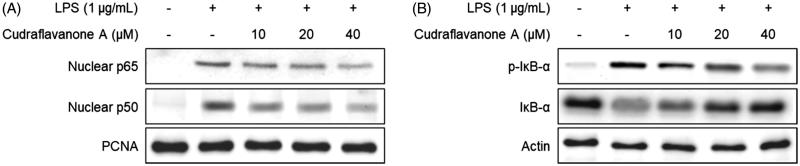
Effect of cudraflavanone A on LPS-induced activation of NF-κB in BV2 cells. Cells were pretreated with the indicated concentrations of cudraflavanone A for 3 h and then stimulated with LPS (1 μg/mL) for 1 h. Nuclear and cytosolic extracts were isolated and the levels of p65 and p50 in the nuclear fraction, and p-IκB-α and IκB-α in the cytosolic fraction were determined by Western blot analysis. PCNA and actin were used as internal controls. The experiment was repeated three times, and similar results were obtained.

To examine the effect of cudraflavanone A on LPS-induced activation of the NF-κB pathway, BV2 cells were pretreated with the indicated concentrations of cudraflavanone A for 3 h, and then stimulated with LPS (1 μg/mL) for 1 h. The cytosolic and nuclear fractions were extracted from the cell lysate, and the protein levels of p65 and p50 were increased in the nuclear fraction of the LPS-treated group. However, the pretreatment of cudraflavanone A prevented the expression of NF-κB subunits in the nuclear fraction (Figure 4(A)). The phosphorylation and degradation of IκB-α in the cytosolic fraction were also increased upon LPS stimulation, but cudraflavanone A inhibited these responses (Figure 4(B)). Therefore, it was inferred that cudraflavanone A exhibited anti-neuroinflammatory effects via suppressing the activation of NF-κB pathway.

### Cudraflavanone A inhibited LPS-induced activation of the MAPK pathways in BV2 cells

**Figure 5.**
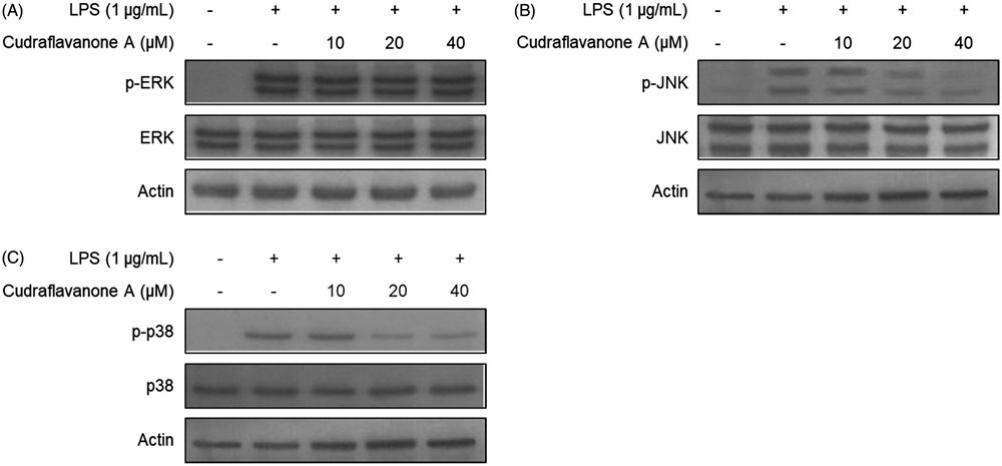
Effects of cudraflavanone A on LPS-induced activation of MAPK pathways in BV2 cells. Lysates were prepared from cells pretreated with/without the indicated concentrations of cudraflavanone A for 3 h and then with LPS (1 μg/mL) for 30 min. The phosphorylated and total forms of p38, ERK and JNK were determined by Western blot analysis. Actin was used as internal controls. The experiment was repeated three times, and similar results were obtained.

It was further investigated whether cudraflavanone A affects the activation of MAPKs pathways. BV2 cells were pretreated with the indicated concentrations of cudraflavanone A for 3 h, and then stimulated with LPS (1 μg/mL) for 30 min. The phosphorylation of ERK, JNK and p38 MAPKs was increased in LPS only treated cells. However, the pretreatment of cudraflavanone A decreased the phosphorylation of JNK and p38 MAPKs (Figure 5(B,C)), but it had no effect on ERK MAPK (Figure 5(A)). These results demonstrate that cudraflavanone A showed anti-neuroinflammatory effects through the JNK and p38 MAPK pathways.

### Cudraflavanone A induced HO-1 protein expression and Nrf2 translocation into the nucleus in BV2 cells

**Figure 6.**
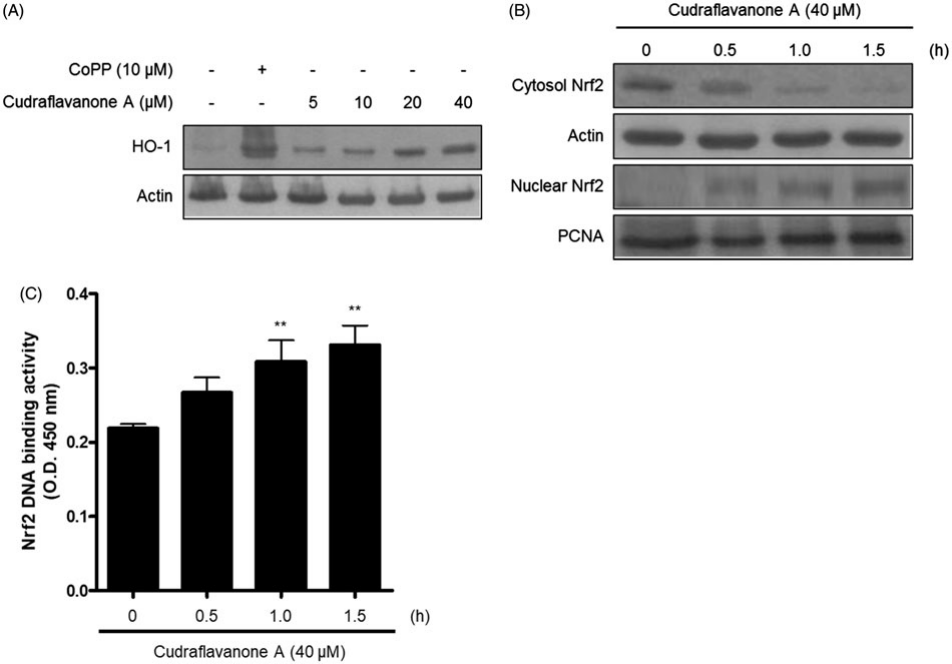
Effects of cudraflavanone A on the HO-1 expression (A), Nrf2 nuclear translocation (B) and Nrf2 DNA binding activity (C) in BV2 cells. (A) Cells were treated with cudraflavanone A for 12 h at various concentrations and Western blot analysis for HO-1 expression was performed. The HO-1 inducer CoPP, was used as a positive control. (B) Cells were treated with 40 μM cudraflavanone A for 0.5, 1 and 1.5 h, and the levels of Nrf2 protein in each fraction were determined by Western blot analysis. (C) Cells were treated with 40 μM cudraflavanone A for 0.5, 1 and 1.5 h. The degree of Nrf2 DNA binding activity was determined by ELISA kit. **p < 0.01 in comparison with 0 h treated group. The experiments were repeated three times and similar results were obtained.

**Figure 7.**
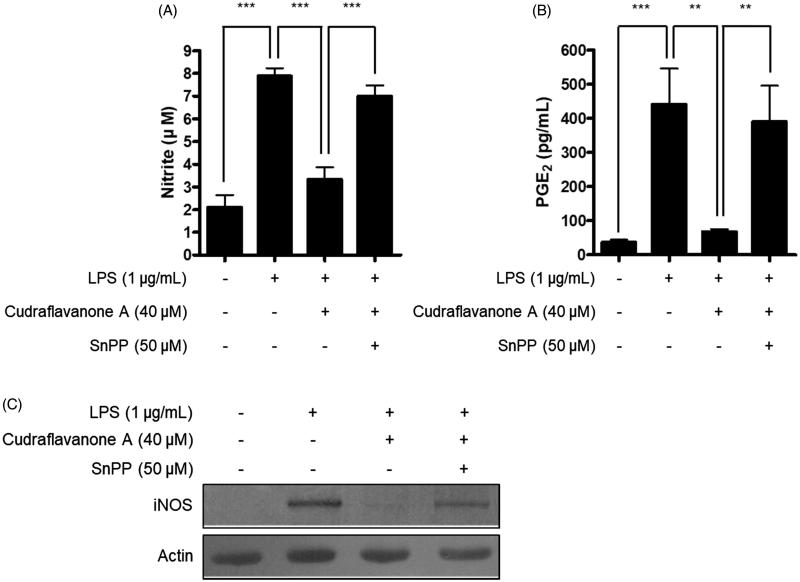
Effects of SnPP on the inhibitory actions of cudraflavanone A on the NO, PGE production and iNOS protein expression. Cells were pretreated with 50 μM of SnPP and incubated with or without cudraflavanone A (40 μM) for 24 h. (A) Nitrite levels were determined using the Griess reaction and (B) PGE_2_ was quantified by ELISA. **p < 0.01 and ***p < 0.001. (C) iNOS protein expression was determined by Western blot analysis, and representative blots from three independent experiments are shown.

Continuously, the correlation between HO-1 expression induced by cudraflavanone A and anti-inflammatory effects of cudraflavanone A was examined. First, whether cudraflavanone A could induce HO-1 protein expression was evaluated by Western blot analysis. Cudraflavanone A induced HO-1 protein expression (Figure 6(A)), and this effect was regulated by cudraflavanone A-mediated nuclear translocation of Nrf2 protein (Figure 6(B,C)). In addition, the influence of SnPP, the established selective HO-1 inhibitor, on the anti-neuroinflammatory effects of cudraflavanone A was examined in LPS-treated BV2 cells. Cudraflavanone A decreased LPS-induced production of NO and PGE_2_, but these effects were partially reversed by pretreatment of SnPP (Figure 7(A,B)). Furthermore, SnPP restored the expression of iNOS protein inhibited by cudraflavanone A (Figure 7(C)). These results indicate that cudraflavanone A exhibited its anti-neuroinflammatory effects through the expression and activity of HO-1 protein.

## Discussion

This study demonstrated that cudraflavanone A appeared to have anti-neuroinflammatory effect against LPS-induced inflammation in BV2 microglial cells. The anti-neuroinflammatory activity of cudraflavanone A was related to the inactivation of NF-κB, and the JNK and p38 MAPK pathways. Moreover, cudraflavanone A exerted its anti-neuroinflammatory effects by inducing HO-1 protein expression and Nrf2 pathway activation.

Activated microglial cells induce the production of pro-inflammatory mediators such as NO, PGE_2_ and pro-inflammatory cytokines as like IL-1β, IL-6 and TNF-α (Zhang et al. 2013). These mediators aggravate and maintain the inflammatory responses (Du et al. 2014). Therefore, the substances which inhibit the release of pro-inflammatory mediators may be valuable for the treatment of neuroinflammatory diseases. In this study, cudraflavanone A was treated in LPS-treated BV2 microglial cells, and it resulted in decreased NO and PGE_2_ production, and inhibition of iNOS protein expression (Figure 2(A–C)). Cudraflavanone A reduced LPS-induced production and expression of IL-1β and TNF-α (Figure 3(A,C,D,F)), but failed to affect IL-6 protein and mRNA expression (Figure 3(B,E)).

Interestingly, cudraflavanone A repressed the production of PGE_2_, but had no effect on COX-2 protein expression and activity. Generally, COX-2 is an important mediator of inflammatory reactions, and COX-2 inhibition has been shown to exert anti-inflammatory (Sano et al. 1992; Yan et al. 2016), and renoprotective effects (Komers et al. 2007; Quilley et al. 2011). However, the long-term treatment with COX-2 inhibitor may induce numerous side effects, including the significant cardiovascular concerns (Jia et al. 2014). Thus, specific agent that targets PGE_2_ synthases may serve as the most promising anti-inflammatory drugs (Trebino et al. 2003; Kudo and Murakami 2005). In particular, microsomal PGE synthase-1 (mPGES-1) is an inducible enzyme essential for the production of pro-inflammatory PGE_2_ from PGH_2_. Under inflammatory conditions, COX-2 converts arachidonic acid to PGH_2_, and eventually mPGES-1 converts PGH_2_ into PGE_2_. Thus, the function of mPGES-1 and COX-2 is coupled to produce PGE_2_ (Oshima et al. 2009). However, studies have shown that mPGES-1 and COX-2 are not always coupled together (Candelario-Jalil et al. 2007; Shie et al. 2015). Therefore, the specific inhibition of mPGES-1 expression or enzyme activity observed in our study may be associated with the suppression of PGE_2_ production by cudraflavanone A.

The genes involved in the production of pro-inflammatory mediators may be regulated by the NF-κB pathway. Under an inactivation state, NF-κB components exist in the cytoplasm with the IκB protein. Upon activation of cells by various stimuli as like LPS, cytokines or chemokines, IκB undergoes phosphorylation and degradation, leading to the translocation of NF-κB components into the nucleus. The translocated NF-κB subunits activate the transcription of pro-inflammatory mediators. Therefore, the inhibition of NF-κB activity may be one of the targets for the treatment of neuroinflammation (Kim et al. 2013). In this investigation, cudraflavanone A attenuated LPS-induced activation of the NF-κB signalling pathway, thereby inhibiting the translocation of NF-κB subunits (p65 and p50), and IκB phosphorylation and degradation (Figure 4). These results suggest that cudraflavanone A could exhibit the anti-neuroinflammatory effects through the inactivation of NF-κB signalling in LPS-stimulated microglial cells.

MAPK pathways consist of three major types, ERK, JNK and p38 MAPKs. These enzymes are related to the extensive activation of pro-inflammatory reactions as well as the NF-κB pathway (Gonzalez-Scarano and Baltuch 1999). In addition, MAPKs are associated with LPS-induced production of pro-inflammatory mediators. Several studies have demonstrated the regulatory role of p38 and JNK MAPKs in inflammatory responses (Da Silva et al. 1997; Guo et al. 2017; Jiang et al. 2017). Results from this study show that cudraflavanone A inhibited the phosphorylation of p38 and JNK MAPKs (Figure 5), and that the p38 and JNK MAPK pathways may be one of the targets for the treatment of neuroinflammation by cudraflavanone A.

The enzyme HO-1 catalyses the degradation of heme, which produces carbon monoxide (CO), ferrous ion and biliverdin. CO can specifically regulate the production of pro-inflammatory cytokines and mediators, and modulate the inflammatory reactions (Ryter and Choi 2016). Nrf2 is the transcription factor that binds to antioxidant response element (ARE)-binding site, activates HO-1 gene, and induces HO-1 expression, leading to anti-inflammatory reactions (Okorji et al. 2016; Yan et al. 2016). Hence, it was investigated whether cudraflavanone A induces HO-1 protein expression and Nrf2 translocation into the nucleus. Treatment of cudraflavanone A induced HO-1 protein expression (Figure 6(A)), and increased the nuclear translocation of Nrf2 in a time-dependent manner (Figure 6(B)). In addition, it was examined whether the anti-neuroinflammatory effect of cudraflavanone A is regulated by HO-1 activity. The pretreatment of cells with SnPP, the established HO activity inhibitor, partially reversed the inhibitory effects of cudraflavanone A on NO and PGE_2_ production, and iNOS protein expression (Figure 7). These results demonstrated that HO-1 expression correlated with the anti-inflammatory effect of cudraflavanone A.

## Conclusions

In this study, cudraflavanone A exerted its anti-neuroinflammatory effects by inhibiting the production of NO and PGE_2_, and expression of iNOS protein. This effect was mediated through the inactivation of NF-κB, as well as the p38 and JNK MAPK pathways in BV2 microglial cells. In addition, cudraflavanone A induced HO-1 expression via Nrf2 translocation into the nucleus, and HO-1 expression was associated with the anti-neuroinflammatory effect of cudraflavanone A. However, cudraflavanone A had no effect on COX-2 protein expression and activity. Taken together, cudraflavanone A may serve as a potent substrate for the development of therapeutic agents to treat neurodegenerative diseases. Therefore, further studies should identify the molecular mechanisms underlying the effects of cudraflavanone A in neuroinflammatory reactions, such as mPGES-1 signalling and upstream pathways of NF-κB or MAPK.
